# Age and Cognitive Ability Predict Perceived Demands of Specific Emotion Regulation Strategies

**DOI:** 10.1093/geroni/igaa057.1471

**Published:** 2020-12-16

**Authors:** Claire Growney, Tammy English

**Affiliations:** 1 North Carolina State University, Raleigh, North Carolina, United States; 2 Washington University in St. Louis, University City, Missouri, United States

## Abstract

Certain emotion regulation (ER) strategies are often considered to be more or less demanding of cognitive resources. However, age-related differences in the perceptions of these demands are not yet understood. Older adults might perceive greater demands for certain strategies due to differences in cognitive ability and motivation to maintain emotional well-being. In the present study, we examined age and cognitive ability as predictors of perceived effort required to use ER strategies that span all families of the process model. A diverse sample of community participants (age 22-83) completed assessments of cognitive ability and perceived demands associated with ten ER strategies. Overall, response-focused strategies were rated as highest in demands whereas situation selection and savoring were perceived as least demanding. Older adults reported higher demands associated with situation selection, distraction, and detached reappraisal (but not positive reappraisal) compared with younger adults. Cognitive ability was not associated with perceived demands for ER strategies traditionally viewed as cognitively demanding (e.g., suppression). Rather, higher cognitive ability only predicted lower perceived demands for strategies often considered low in demand: situation selection and savoring. Perceived ER success was not consistently associated with age or cognitive demands. Results suggest that older adults view some, but not all, ER strategies as more demanding than younger adults do. The role of cognitive ability in age-related changes in ER may be more complex than previously expected. Notably, the lack of findings with perceived ER success suggest effort requirements associated with ER may not impede ability to successfully regulate across adulthood.

